# The Awareness of the Jordanian Population Regarding Intranasal Corticosteroids' Uses for Allergic Rhinitis

**DOI:** 10.1055/s-0045-1810049

**Published:** 2025-10-16

**Authors:** Hasan Ibrahim Al-Balas, Almu'atasim Khamees, Tala Abd Aljaleel Okour, Ruaa Ismail Ababneh, Bara' Hussein Al-Smadi, Leen Mohamad Almomani, Reem Nazem BaniHani, Marah Akram AlRawashdeh

**Affiliations:** 1 Department of Otorhinolaryngology, Faculty of Medicine, Yarmouk University, Irbid, Jordan; 2 Department of General Surgery, Princess Basma Teaching Hospital, Ministry of Health, Irbid, Jordan; 3 Department of Clinical Science, Faculty of Medicine, Yarmouk University, Irbid, Jordan

**Keywords:** allergic rhinitis, intranasal corticosteroids, awareness, knowledge

## Abstract

**Introduction:**

Intranasal corticosteroids are widely regarded as the most efficacious remedy for managing moderate to severe allergic rhinitis. However, the Jordanian population's understanding and attitudes toward the safe and proper use of these medications remain ambiguous.

**Objective:**

This study was undertaken to evaluate Jordanian individuals' awareness of intranasal corticosteroids by examining their present perceptions, attitudes, and behaviors regarding these pharmaceuticals.

**Methods:**

We conducted a cross-sectional study within the adult Jordanian demographic using an online survey based on a structured questionnaire.

**Results:**

The study comprised 1509 participants, revealing that 34.2% of the adult Jordanian population suffers from allergic rhinitis, with a prevalence of 39.5% in males and 60.5% in females. Among individuals with allergic rhinitis, 34.1%, 38.4%, 67.1%, and 43.2% hold beliefs regarding the impact of intranasal corticosteroids on blood sugar, blood pressure, pregnancy, and obesity, respectively. Notably, 67.8% of allergic rhinitis patients in our study actively use intranasal corticosteroids, and users demonstrate a significantly higher level of awareness compared with non-users, as we found significantly higher awareness levels among allergic rhinitis patients regarding possible side effects compared with non-allergic rhinitis group (p-value = < .0001, 0.0019, 0.0109, <.0001 and 0.0034) for blood sugar, blood pressure, pregnancy, children less than 5 years and obesity respectively.

**Conclusion:**

The results obtained from this research underscore the critical need for educational initiatives and interventions to enhance understanding and utilization of these medications within Jordan. These efforts can significantly contribute to improved patient outcomes and decreased healthcare expenses.

## Introduction

Allergic rhinitis is characterized by inflammation of the nasal mucosa induced by exposure to allergens. 1 This prevalent global health issue impacts millions of people, with a worldwide prevalence ranging from 10% to 30% of the population. 2 It significantly affects the quality of life and productivity of those affected. 3 Although official data on the prevalence of allergic rhinitis are not available, estimates of at least 15–20% of Jordanians afflicted with the condition make it a significant public health concern in Jordan. 4 This condition is characterized by symptoms such as sneezing, nasal congestion, nasal itching, and rhinorrhea (nasal discharge). 5 When left uncontrolled, these symptoms can lead to sleep disturbances, resulting in daytime fatigue, learning difficulties, decreased cognitive functioning, reduced long-term productivity, and an overall decline in the quality of life. 6

Both medical and non-medical treatment options are available for allergic rhinitis. Non-medical approaches involve allergen avoidance, while medical treatments encompass antihistamines, steroids, decongestants, chromones, and immunotherapy. 7 Among these, intranasal corticosteroids are a widely employed therapeutic choice for effectively managing the symptoms of allergic rhinitis. 8 These corticosteroids, known for their potent anti-inflammatory properties, directly target the nasal mucosa, quelling the inflammatory response induced by allergens, and delivering enduring relief from symptoms like nasal congestion, sneezing, and nasal itching. 9 It is essential to note that using intranasal corticosteroids (INCS) is considered safe, with occurrences of nasal septal perforation being extremely rare. Local side effects, such as mucosal irritation and epistaxis, are typically minor. 9

Despite the prevalent use of intranasal corticosteroids for treating allergic rhinitis, a knowledge gap exists among patients regarding the correct utilization of these medications. This lack of awareness can lead to misuse or underuse of these drugs, impacting patient care and increasing healthcare costs. 10 Also, misconceptions about INCS causing systemic side effects, such as elevated blood sugar, hypertension, or harm during pregnancy, are prevalent, often rooted in conflation with oral steroids. Additionally, some patients erroneously associate INCS with obesity and avoid them entirely due to unfounded fears of addiction or nasal damage. 11 Thus, it is imperative to assess the level of understanding within the Jordanian population concerning the appropriate use of intranasal corticosteroids for allergic rhinitis.

This research endeavors to gauge the Jordanian population's awareness regarding using intranasal corticosteroids for allergic rhinitis. It aims to bridge this knowledge gap by exploring the current perceptions, attitudes, and practices of Jordanian individuals toward these medications. Additionally, this study will delve into the factors influencing the utilization of these medications, including age, gender, educational background, and socioeconomic status. The insights gained from this research could inform the development of educational programs and interventions, fostering improved knowledge and appropriate usage of these medications in Jordan. Ultimately, this endeavor seeks to enhance patient outcomes and curtail healthcare costs.

## Methods

This study adopted a cross-sectional design employing an online self-administered questionnaire targeting Jordanian adults aged 18 years and above. The authors developed a validated and reliable questionnaire before carrying out the current study, as the first aim of this project was to develop and validate the questionnaire, aiming to develop an instrument tool to assess the awareness of the Jordanian population regarding the usage of intranasal corticosteroids for allergic rhinitis. The questionnaire was designed based on a comprehensive review of existing literature on allergic rhinitis (AR) and intranasal corticosteroids (INCS), including validated tools from prior studies. 11 12 Key domains (knowledge, attitudes, practices, and demographic factors) were identified through consensus among the research team. A pilot study involving 30 participants (not included in the final sample) was conducted to assess clarity, comprehensibility, and cultural relevance. Feedback revealed minor ambiguities in phrasing, which were subsequently revised. Cronbach's α was calculated for multi-item scales (e.g., awareness of side effects), yielding values ranging from 0.72 to 0.84, indicating acceptable to good internal consistency. After that, content validity was ensured through expert review by three independent clinicians specializing in allergy and immunology. Items were rated for relevance on a 4-point scale, with an average content validity index (CVI) of 0.89, exceeding the recommended threshold of 0.78.

The questionnaire was distributed on August 4th, 2023, utilizing various social media platforms, the University's library email, and direct personal connections. The questionnaire comprised multiple-choice questions and obtained institutional review board approval via (***) University's office (IRB/2023/276). Participants were briefed on the objectives and purpose of the questionnaire, with implied consent upon completion. Data analysis utilized the Statistical Package for the Social Sciences (SPSS) version 27, involving descriptive statistics and chi-square testing, with statistical significance set at *p* < 0.05. The chi-square test was selected to evaluate associations between categorical variables. This method is appropriate for testing independence between nominal variables in our cross-sectional design. Our primary aim was to compare the proportions of awareness between groups (INCS users versus non-users), and chi-square tests are widely accepted for this purpose in similar epidemiological studies.

## Results

The questionnaire encompassed 1509 participants, with 34.2% reporting allergic rhinitis and 65.8% being free from it. Data collection involved 562 males and 947 females, with 39.5% of males and 60.5% of females experiencing allergic rhinitis. Among those with allergic rhinitis, 25.6% were smokers, while 74.4% were non-smokers. Participants with allergic rhinitis detailed symptoms like rhinorrhea, congestion, cough, headache, fatigue, eye itching, ear fullness, and post-nasal drip. ( Table 1 ).

**Table 1:** Symptoms for patients with allergic rhinitis

SYMPTOM	MALE	FEMALE	TOTAL
Rhinorrhea	61 (29.9%)	109 (34.9%)	170 (32.9%)
Congestion	135 (66.2%)	96 (30.8%)	231 (44.8%)
Cough	86 (42.2%)	157 (50.3%)	243 (47.1%)
Nasal itching	85 (41.7%)	156 (50%)	241 (46.7%)
Headache	83 (40.7%)	186 (59.6%)	269 (52.1%)
Fatigue	63 (30.9%)	157 (50.3%)	220 (42.6%)
Ocular itching	52 (25.5%)	128 (41%)	180 (34.9%)
ear fullness	42 (20.6%)	134 (42.7%)	176 (34.1%)
Post nasal drip	68 (33.3%)	131(42%)	199 (38.6%)

Assessment of awareness about nasal corticosteroid side effects revealed that 43.2% of non-allergic rhinitis participants and 34.1% of allergic rhinitis participants believed it affects blood sugar (P value < 0.05). Similarly, 46.7% of non-allergic rhinitis and 38.4% of allergic rhinitis individuals thought it affected blood pressure (P value < 0.05). Concerning pregnancy, 73.3% of non-allergic rhinitis and 67.1% of allergic rhinitis participants believed corticosteroids affect it (P value < 0.05). About obesity, 51.2% of non-allergic rhinitis and 43.2% of allergic rhinitis individuals held that belief (P value < 0.05). No statistically significant value among other parameters regarding the awareness among both allergic rhinitis and non-allergic rhinitis patients ( Table 2 )

**Table 2:** Awareness regarding intranasal corticosteroids among those without allergic rhinitis and those with allergic rhinitis

	FREE		AR	
	YES	NO	YES	NO
Addiction	391(39.4%)	602(60.6%)	182(35.3%)	334(64.7%)
Blood sugar	429(43.2%)	564(56.8%)	176(34.1%)	340(65.9%)
Blood pressure	464(46.7%)	529(53.3%)	198(38.4%)	318(61.6%)
Pregnancy	728(73.3%)	265(26.7%)	346(67.1%)	170(32.9%)
Affects Childs less than 5 years	825(83.1%)	168(16.9%)	370(71.7%)	146(28.3%)
Osteoporosis	402(40.5%)	591(59.5%)	184(35.7%)	332(64.3%)
Hormonal disturbances	480(48.3%)	513(51.7%)	235(45.5%)	281(54.5%)
Obesity	508(51.2%)	485(48.8%)	223(43.2%)	293(56.8%)
Acne	356(35.9%)	637(64.1%)	164(31.8%)	352(68.2%)
TOTAL	993		516	

In evaluating intranasal corticosteroid usage among allergic rhinitis patients, 67.8% actively used them while 32.2% did not. Interestingly, users demonstrated higher awareness levels than non-users. Notably, the percentages of those believing in adverse effects like blood sugar, blood pressure, obesity, hormonal disturbances, and acne were lower among users (29.4%, 34%, 38.6%, 41.7%, and 26%, respectively) compared with non-users within the allergic rhinitis group with statistic significant P value (P value < 0.05) ( Table 3 ).

**Table 3:** Awareness regarding intranasal corticosteroids between those who use or not among AR patients

	AR / USE SPRAY		AR / NOT USE	
	YES	NO	YES	NO
Addiction	119(34%)	231(66%)	63(38%)	103(62%)
Blood sugar	103(29.4%)	247(70.6%)	73(44%)	93(56%)
Blood pressure	119(34%)	231(66%)	79(47.6%)	87(52.4%)
Pregnancy	227(64.9%)	123(35.1%)	119(71.7%)	47(28.3%)
Child less than 5 years	249(71.1%)	101(28.9%)	121(72.9%)	45(27.1%)
Osteoporosis	120(34.3%)	230(65.7%)	64(38.6%)	102(61.4%)
Hormonal disturbances	146(41.7%)	204(58.3%)	89(53.6%)	77(46.4%)
Obesity	135(38.6%)	215(61.4%)	88(53%)	78(47%)
Acne	91(26%)	259(74%)	73(44%)	93(56%)
TOTAL	350(67.83%)		166(32.17%)	

## Discussion

In Jordan, as in numerous other nations, allergic rhinitis stands as a notable public health concern, affecting up to 20% of the general populace, thereby exerting a substantial influence on the affected individuals' quality of life and overall productivity. 4 Recent epidemiological studies conducted in the Kingdom of Saudi Arabia (KSA) have elucidated that the prevalence of allergic rhinitis (AR) varies within the range of 15% to 40%, exhibiting variations contingent upon factors such as age, gender, and other sociodemographic attributes. 13 14

There are many cultural barriers in Middle Eastern populations, such as the usage of corticosteroids, which is often stigmatized due to misconceptions about systemic side effects like weight gain or hormonal imbalances, which are more commonly associated with oral steroids. This conflation likely contributes to reluctance toward INCS adoption. Also, Traditional practices, such as reliance on herbal remedies, are prevalent for managing AR symptoms. In addition to the throat rundown and aftertaste from INCS, reduces compliance, especially with frequent dosing. These practices, rooted in cultural heritage, may divert patients from evidence-based treatments like INCS or reduce INCS adherence. 11 15 16

Nasal corticosteroids, often called INCSs, are a crucial treatment for people dealing with moderate to severe Allergic Rhinitis (AR), and this recommendation comes from established medical guidelines. 9 17 The way patients follow their prescribed treatment is really important because it directly affects how well they feel and how their symptoms improve. However, sometimes, patients get worried about possible side effects, and this fear can lead them to stop their treatment earlier than they should. This not only costs them more money but also makes their overall quality of life worse. 10 18 So, it's vital to make sure patients have good information about the safety of these nasal sprays, helping them set realistic expectations and choose the right treatment for themselves. Moreover, Rifaei SM et al found that only 40.9% of Jordanian patients receive side-effect information from providers, which emphasizes the need for pharmacist-led education. 15

Our research has revealed a substantial deficiency in the awareness of the Jordanian population concerning the advantages of intranasal corticosteroids for managing allergic rhinitis. This knowledge gap becomes apparent when considering the high proportion of respondents in our survey who acknowledged never having utilized these medications, despite their well-documented efficacy in alleviating symptoms. This discovery prompts pertinent inquiries regarding the accessibility of information to the general public pertaining to treatments for allergic rhinitis. Furthermore, it underscores the pressing need for comprehensive public health campaigns and proactive interventions by healthcare providers to enhance the populace's understanding of the potential benefits of intranasal corticosteroids in improving their quality of life and effectively managing allergic rhinitis symptoms. About one-third of the sample has AR, and results indicate that participants with allergic rhinitis have shown more awareness regarding intranasal corticosteroids in comparison with those who don't have allergic rhinitis. An anticipated result, knowing intranasal corticosteroids are a superior line of treatment for AR. 19

Among the parameters used to assess the side effects of intranasal corticosteroids, the awareness includes blood sugar, blood pressure, obesity, and pregnancy. These previously mentioned parameters have shown a significant difference between the two groups. Similarly, in a study conducted in Saudi Arabia that showed participants with AR,88.5%, 92%, and 88% of participants believed that high blood pressure, high blood sugar, and obesity are not side effects of the usage of intranasal corticosteroids. 11 Also, studies consistently show low public awareness of INCS safety, particularly regarding systemic side effects. 11 15

As previously mentioned, patients with allergic rhinitis have shown more awareness regarding intranasal corticosteroids. However, in our study, we found that almost one-third of allergic rhinitis patients did not use intranasal corticosteroids, as those patients have shown significant knowledge gaps about intranasal corticosteroids' side effects in specific parameters, including blood sugar, blood pressure, obesity, hormonal disturbances, and acne. A study was conducted in Riyadh, Kingdom of Saudi Arabia; that study found that most of the participants did not think that high blood pressure, high blood sugar, and obesity were side effects of using steroids. 11 Our results showed that patients who choose not to use INCSs show significantly different expectations and concerns regarding INCS's potential side effects compared with those who choose to use INCSs. 29.4% and 44% have concerns about INCS's effect on blood sugar among INCS users and non-users, respectively.

Similar significant concerns were found regarding blood pressure for 34%, and 47.6%, obesity for 38.6%, and 53%, hormonal disturbances for 41.7%, and 53.6%, and acne for 26%, and 44% among INCS users and non-users respectively. Similar findings were reported in a previous study conducted in Australia, which found that 67% of the patients had little or no knowledge about INCSs. 12 This significant difference suggests that patient awareness about potential INCS side effects has an important impact on patients' usage of these drugs.

Finally, culturally tailored and institutionally supported interventions are critical to addressing gaps in intranasal corticosteroid awareness in Jordan. First, in collaboration with local NGOs like the Jordanian Medical Association, the Jordanian Ministry of Health should develop Arabic-language educational materials that address widespread misconceptions (for example, INCSs causing systemic side effects like obesity) using relatable analogies and visual aids tailored to low-literacy populations. These materials could be distributed via primary care clinics, pharmacies, and community centers. Second, training programs for pharmacists and primary care providers should be prioritized, emphasizing INCS safety and correct administration techniques. Third, leveraging Jordan's high social media penetration, targeted campaigns on platforms like Facebook and WhatsApp, featuring trusted figures, could debunk myths and promote INCS benefits. Fourth, integrating INCS education into school health curricula could normalize their use among families. Fifth, establishing a national allergic rhinitis awareness month by partnering with Jordan University Hospital for free screening camps would improve accessibility and visibility. Finally, integrating INCS affordability into Jordan's national health insurance scheme and subsidizing costs for low-income populations would mitigate financial barriers. These steps, grounded in Jordan's sociocultural and infrastructural realities, offer a roadmap to bridge knowledge gaps, reduce AR burden sustainably, reduce preventable morbidity, and alleviate long-term healthcare costs.

## Strengths of the Study

This study offers several key strengths that enhance its contribution to understanding intranasal corticosteroid (INCS) awareness in Jordan. First, the large, nationally representative sample ( *N* = 1,509) provides robust statistical power and improves the generalizability of findings across diverse sociodemographic groups, including urban and rural populations. Second, the focus on region-specific misconceptions—such as fears of systemic side effects (e.g., 67.1% associating INCS with pregnancy risks) and avoidance due to unfounded concerns (32.2%)—fills a critical gap in Middle Eastern literature, where cultural stigma around corticosteroids is understudied. Third, the comparative analysis between INCS users and non-users revealed actionable insights, demonstrating significantly higher awareness among users ( *p* < 0.001), underscoring the role of healthcare engagement in dispelling myths. Using validated statistical methods (chi-square tests) strengthened the reliability of associations. Finally, the findings directly inform scalable, culturally tailored interventions, such as pharmacist-led education and social media campaigns, offering a roadmap to reduce allergic rhinitis burden in Jordan. These strengths collectively enhance the study's relevance to public health policy and clinical practice in similar settings.

## Limitations and Recommendations for Future Research

This study has several limitations. First, the reliance on an online, self-administered questionnaire may introduce sampling bias, as it excludes individuals without internet access or digital literacy, potentially underrepresenting rural, elderly, or socioeconomically disadvantaged populations. Second, the absence of validation details for the questionnaire (e.g., pilot testing, reliability metrics, or cultural adaptation) raises concerns about its accuracy and relevance to the Jordanian context. Third, while the cross-sectional design captures awareness at a single point in time, it cannot establish causality or track changes in perceptions over time. Additionally, the study did not assess clinical data, such as AR severity or appropriateness of INCS prescriptions, limiting insights into adherence alignment with treatment guidelines. However, to address the limitations of this study, future research should adopt a mixed-methods approach combining quantitative surveys with qualitative interviews to capture nuanced cultural and socioeconomic factors influencing INCS awareness. Community-based recruitment strategies, including in-person data collection in rural and underserved areas, could mitigate sampling bias and improve representativeness. Validating the questionnaire through pilot testing, expert review, and cross-cultural adaptation would enhance its reliability and relevance. Longitudinal designs could track changes in awareness and adherence over time, while multivariate analyses (e.g., logistic regression) should explore predictors such as education, income, and healthcare access. Integrating clinical data (e.g., AR severity, prescription patterns) with patient-reported outcomes would clarify the alignment between awareness and treatment guidelines. Collaborations with healthcare providers could further contextualize findings and inform targeted interventions, ensuring equitable educational strategies for diverse populations.

## Conclusion

The results from this research underscore the critical need for educational initiatives and interventions to enhance understanding and utilization of these medications within Jordan. By doing so, these efforts can significantly contribute to improved patient outcomes and decreased healthcare expenses.
